# In Human Monocyte Derived Dendritic Cells SOCS1 Interacting with CYTIP Induces the Degradation of CYTIP by the Proteasome

**DOI:** 10.1371/journal.pone.0057538

**Published:** 2013-02-28

**Authors:** Daniela Grabher, Susanne Hofer, Daniela Ortner, Christine Heufler

**Affiliations:** 1 Department of Dermatology, Innsbruck Medical University, Innsbruck, Austria; 2 Department of Gynecological Endocrinology and Reproductive Medicine, Innsbruck Medical University, Innsbruck, Austria; Temple University, United States of America

## Abstract

CYTIP (cytohesin interacting protein) is an intracellular molecule induced in dendritic cells during maturation. CYTIP modulates the binding intensity of the adhesion molecule LFA-1. If dendritic cells are silenced for CYTIP they keep longer contacts with T-cells resulting in a lower T cell stimulation. We identified Suppressor of cytokine signaling-1 (SOCS-1) as a binding partner for CYTIP in human monocyte derived dendritic cells. In Western blot analyses we found that CYTIP expression is down regulated at later time points, starting at about 72 hours after induction of maturation. To investigate a possible role for SOCS-1 in taking CYTIP to the degradation machinery of the cell we measured endogenous CYTIP protein levels in mature dendritic cells transfected with SOCS-1 encoding plasmid in quantitative Western blot analyses. We observed lower amounts of endogenous CYTIP in mature dendritic cells transfected with SOCS-1 encoding plasmid compared with untransfected dendritic cells. Experiments with the proteasome-inhibitor Bortezomib/Velcade® show stable CYTIP expression levels in dendritic cells. In addition, we show that CYTIP in dendritic cells matured for 48 hours is ubiquitinated and thus ready for degradation. We here describe a newly identified binding partner of CYTIP, SOCS-1, and confirm its function in regulating the degradation of CYTIP by the proteasome.

## Introduction

Dendritic cells reside in tissues exposed to the environment such as the skin and the mucosa of the airways and the gut where they take up samples of the incoming proteins. These proteins are processed and presented on major histocompatibility complex (MHC) class I and class II molecules and can be recognized by the adaptive immune system via T cell receptors in lymphoid tissues [1]. This recognition is a key event in the initiation of adaptive immune responses and the conditions encountered here are decisive for the T cell subtype induced, e.g. TH1, TH2, TH17 or Treg, and, thus, the outcome of the immune response. Therefore, regulating the interaction of dendritic cells with T cells is an important checkpoint in the initiation of adaptive immune responses. This interaction has been shown to be short lived during the scanning process while searching for antigen specificity of the T cell receptor for the antigen presented on MHC. It changes to a stabile interaction if specificity is encountered to allow for T cell activation and followed by detachment of the T cells for proliferation and migration to the entry site of the antigen [2]–[5]. We and others have shown that cytohesin interacting protein (CYTIP) (Cybr, CASP) regulates dendritic cell - T cell interactions in humans by modulating the adhesion molecule LFA-1. LFA-1 is long known to be expressed on dendritic cells and its role in T cell activation was resolved, when the activation status of LFA-1 was shown to regulate contact duration with T cells [6]. Boehm et al. [7] found CYTIP expression to be induced in mature dendritic cells and identified CYTIP as interaction partner for cytohesin-1. Cytohesin-1 activates LFA-1 by binding to the intracellular part of CD18, the ß chain of LFA-1. CYTIP interacting with cytohesin-1 keeps cytohesin-1 from binding to CD18, thus inhibiting LFA-1 activation. Hofer et al. [8] showed that CYTIP expression is necessary for correct detachment of T cells from dendritic cells during immune responses and showed that inhibition of CYTIP expression in dendritic cells by siRNA leads to impaired T cell activation. Inhibition of CYTIP expression is also induced by herpes simplex virus (HSV) infection of dendritic cells, with consequences on their ability to migrate and to interact with the interstitial space and T cells. Theodoridis et al [9] found that CYTIP is rapidly degraded in dendritic cells infected with HSV leading to impaired migration of dendritic cells and impaired immune responses. This is thought to be a novel viral evasion mechanism from immune responses. The degradation of CYTIP induced by the virus was found to be independent of virion host shutoff protein (vhs) or the immediate early protein IP10 but mediated by the proteasome. Here we describe SOCS-1 as a newly identified interaction partner for CYTIP. SOCS-1 is involved in many different cytokine pathways and has a wide range of activities. One major function of this molecule is the negative regulation in the signaling cascade of many cytokine receptors. SOCS-1 targets proteins involved in these signaling pathways and brings them to the proteasome for degradation [10], [11]. From the data described above we deduced the hypothesis that binding of SOCS-1 to CYTIP might take CYTIP to the proteasome for degradation. Here we present evidence supporting this hypothesis.

## Materials and Methods

### Generation of Monocyte-derived Dendritic Cells

Human dendritic cells were prepared from peripheral blood monocytes as described [12], [13]. Anonymous human blood components were obtained from the local blood bank (Central Institute for Blood Transfusion and Immunology, Innsbruck Medical University, Innsbruck, Austria) according to the guidelines of the local blood bank approved by the independent ethics committee of the Innsbruck Medical University and the tenets of the Helsinki Protocol.

### Yeast Two-hybrid Screening

To identify binding partners of CYTIP its coding region was cloned into the bait vector and used to screen a cDNA library prepared from mature monocyte derived dendritic cells using the Yeast two hybrid library preparation kit (Clontech, Palo Alto, CA) with the GAL4-based Matchmaker Two-hybrid system 2 (Clontech, Palo Alto, CA) according to the manufacturer’s protocol.

### Co-immunoprecipitation

5×10^6^ mature dendritic cells were lysed in 0.5 ml of modified RIPA buffer supplemented with 2 µg/ml aprotinin, 10 µg/ml leupeptin, and 1 µg/ml pepstatin A. Protein L agarose was used to pre-clear the lysate. 2 mg of protein lysate were incubated with rat polyclonal anti CYTIP antibody (1A3) and incubated for 4 h at 4 C. 100 µl of Protein L agarose were added overnight at 4°C. The immunocomplex was obtained by centrifugation and released from the protein L agarose by adding 30 µl of modified RIPA buffer, 10 µl of loading puffer and heating at 95°C for 5 minutes. After centrifugation the supernatant was collected to be analyzed for the presence of SOCS-1 or Ubiquitin by Western blot analyses.

### Western Blot Analysis

Cells were lysed in RIPA buffer supplemented with 2 µg/ml aprotinin, 10 µg/ml leupeptin, and 1 µg/ml pepstatin A, and subjected to SDS-PAGE. CYTIP was detected using the CYTIP-specific antibody 1A3 followed by Alexa fluor 680 goat anti rat IgG (Invitrogen, Eugene, Oregon, USA). SOCS-1 was detected with the rabbit polyclonal anti-hu SOCS-1 antibody (Imgenex San Diego, Ca, USA and GeneTex, Irvine CA, USA) followed by Alexa Fluor 680 goat anti-rabbit IgG (Invitrogen, Eugene, Oregon, USA). Ubiquitin was detected with the mouse monoclonal anti ubiquitin antibody (Santa Cruz Biotechnology, Santa Cruz, CA, USA) and visualized with Alexa fluor 680 goat anti mouse antibody (Invitrogen, Eugene, Oregon, USA). The membranes were scanned using Odyssey Infrared Imaging System (LiCor Biosciences, Lincoln, NE, USA).

### Transfection of Mature Dendritic Cells

For the transfection of mature dendritic cells we used Amaxa Nucleofection technology according to the manufacturer’s protocol. After transfection, cells were plated into the prepared medium in 12-well plates and maturation cocktail (TNFα 10 ng/ml, IL-1β 2 ng/ml, IL-6 1000 U/ml and PGE2 1 µg/ml) was added.

### Bortezomib (Velcade®) Treatment of Dendritic Cells

At day 6 of culture, dendritic cells were matured as described before and simultaneously treated with Bortezomib at a concentration of 5 nM for 48 hours. For experiments in which Bortezomib treated cells needed to be transfected, cells had to be harvested after 24 hours of maturation and treatment with Bortezomib for transfection and analyses were done after additional 16 hours of incubation.

### FACS Analysis of Dendritic Cells Treated or not with 5 nM Bortezomib

Dendritic cells treated or not with 5 nM Bortezomib during maturation were collected and stained with FITC labeled anti human CD80, -CD83, -CD86, -HLADR and HLA A,B,C (Becton-Dickinson, NJ, USA) antibodies. Cells were analyzed on the FACS Calibur (Becton-Dickinson) and data handled with FLOWJO software.

## Results

### CYTIP Binds to SOCS-1 in Mature Dendritic Cells

**Figure 1:**
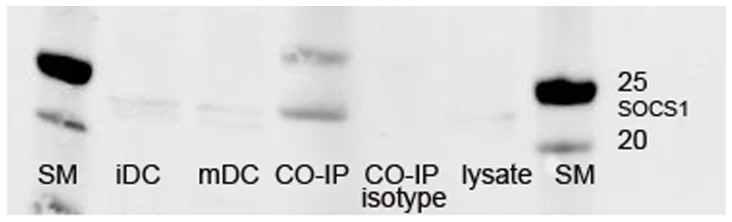
Co-immunoprecipitation of SOCS-1 with CYTIP. Rat polyclonal anti CYTIP antibody (1A3) was used to co-immunoprecipitate CYTIP and its binding partners in 2 mg of mature dendritic cells lysate. Rabbit anti human SOCS-1 polyclonal antibody was used for detection and visualized with Alexa fluor 680 goat anti rabbit antibody. SOCS-1 expression in immature (iDC) and mature dendritic cells (mDC) and in the immunoprecipitate (CO-IP) is shown. As a control, rat IgG1 Isotype control was used for co-immunoprecipitation (Isotype control). SM: size marker.

CYTIP contains several protein-protein binding sites but only cytohesin-1 [14] binding to the coiled coil domain and sorting nexin 27 (SNX 27) [15] binding to the PDZ binding motif have been characterized so far. This makes the existence of additional binding partners likely. We therefore chose to further evaluate the function of CYTIP by searching for an additional binding partner of CYTIP in mature dendritic cells. In a yeast-two-hybrid system with CYTIP as bait we screened a library derived from mature monocyte derived dendritic cells. As expected, the most frequent sequence obtained coded for cytohesin-1, which was identified as binding partner for CYTIP earlier [7]. The second most frequent sequence of the isolated clones coded for SOCS-1. To verify our result from the yeast-two-hybrid screen we co-immunoprecipitated the CYTIP-SOCS-1 complex from protein lysate derived from mature monocyte derived dendritic cells with monoclonal rat anti CYTIP antibody 1A3 and detected SOCS-1 in Western blot analyses using polyclonal rabbit anti human SOCS-1 antibody (Fig. 1 CO-IP). As a control, the co-immunoprecipitation was carried out with an isotype matched control antibody (isotype control). In addition, SOCS-1 expression in immature (iDC) and mature dendritic cells (mDC) and in the lysate used for co-immunoprecipitations is shown.

### CYTIP Expression Increases during Maturation and Decreases at Later Time Points While SOCS-1 Expression Remains Stable

**Figure 2:**
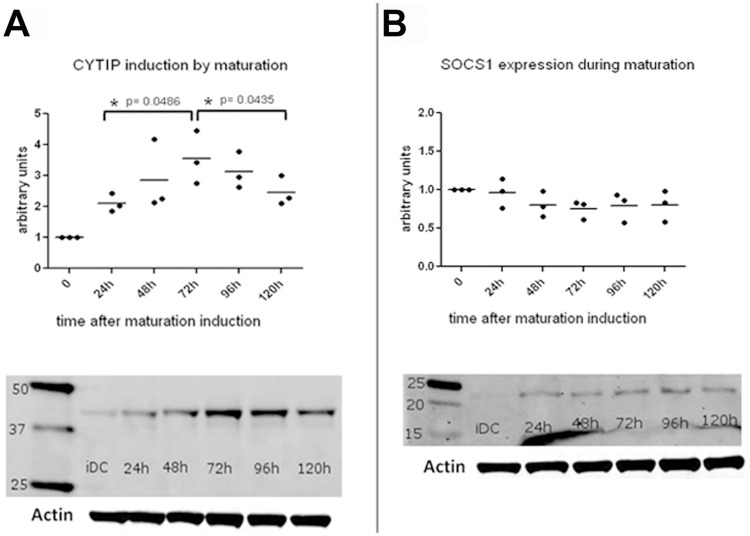
CYTIP and SOCS-1 expression levels at different time points during maturation in monocyte derived dendritic cells. (A) CYTIP expression levels during maturation of dendritic cells were measured at the indicated time points. Western blots were performed using rat anti human CYTIP antibody 1A3 and visualized with Alexa fluor 680 goat anti rat antibody. Three independent Western blot analyses were analyzed and show a peak expression for CYTIP at 72 hours after induction of maturation. One representative Western blot is shown. (B) SOCS-1 expression during maturation remains stable over time. Western blot analyses were performed with lysates harvested at the indicated time points using rabbit polyclonal anti SOCS-1 antibody and visualized with Alexa fluor 680 goat anti rabbit antibody. One representative Western blot is shown. Arbitrary units were set to show progression of CYTIP (A) and SOCS-1 (B) expression during maturation. Immature dendritic cells (iDC) were used as reference level at time point 0 of maturation.

To evaluate expression levels over time, dendritic cells were matured at day 6 of culture and harvested 0, 24, 48, 72, 96 and 120 hours after maturation induction. Lysates of all time points were analyzed on Western blots for CYTIP and SOCS-1 levels. In three independent experiments we show that CYTIP expression levels increase during maturation up to 72 hours and decrease afterwards (Fig. 2A) while SOCS-1 expression levels remain constant (Fig. 2B). Statistical analysis was performed using paired, two-tailed student’s t-test. One representative Western blot each is shown with actin levels as loading control.

### Endogenous CYTIP Expression Decreases When SOCS-1 is Over Expressed

**Figure 3:**
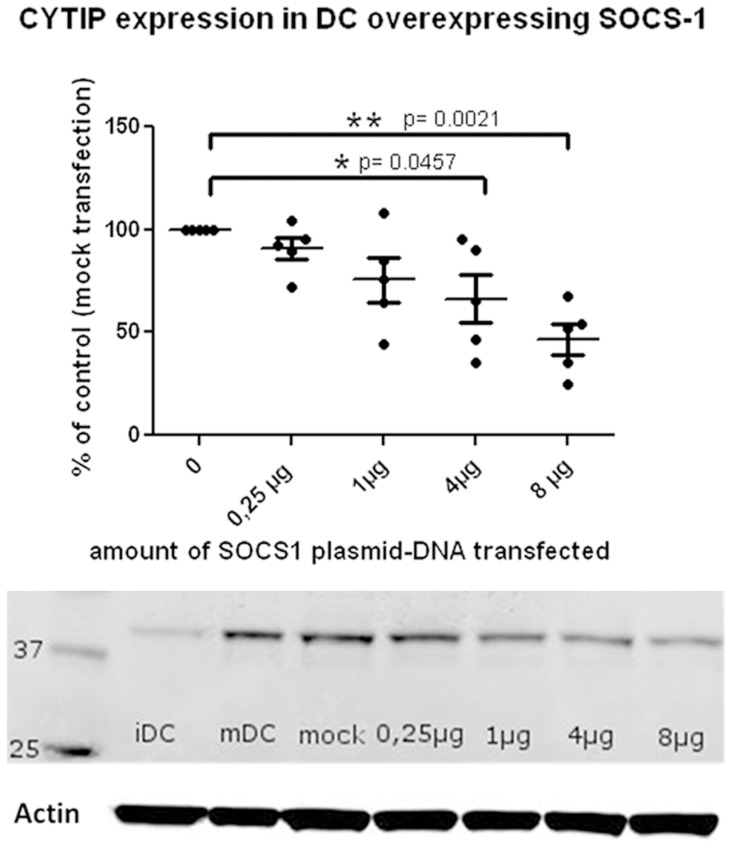
Transfection of mature dendritic cells (mDC) with increasing amounts of SOCS-1 encoding plasmid induces a decrease of CYTIP expression. 1×10^6^ cells were transfected with the indicated amounts of SOCS-1 encoding plasmid. 16 hours later protein lysates were prepared and 40 µg of lysates were applied to each lane. CYTIP levels (39 kDa) were measured by Western blot analyses. As a control, actin expression levels were determined. Quantification of grey level pixel intensity was done with Odyssey software. One representative Western blot is shown. iDC: immature dendritic cells, mDC: mature dendritic cells, mock: transfection procedure without plasmid DNA, µg indicate the amount of plasmid used for transfection. CYTIP expression decreases significantly with SOCS-1 over expression 16 hours after transfection with 4 and 8 µg of SOCS-1 encoding plasmid.

To evaluate whether CYTIP is degraded as a consequence to its binding to SOCS-1 we measured CYTIP expression levels in mature dendritic cells transfected with increasing amounts of a SOCS-1 expression vector. If so, increasing amounts of transfected SOCS-1 should decrease the amount of CYTIP protein in dendritic cells. We used the indicated amounts of SOCS-1 encoding plasmid to transfect 1×10^6^ mature dendritic cells each. 16 hours after transfection Western blot analysis with anti CYTIP antibody was performed. As shown in Fig. 3, in 5 independent experiments we found that CYTIP expression decreases with growing amounts of transfected SOCS-1 expression vector. Statistical analysis was performed using paired, two-tailed student’s t-test. Expression levels obtained with 4 and 8 µg of SOCS-1 plasmid DNA transfection resulted in significant decrease of CYTIP expression. One representative Western blot with immature and mature dendritic cells, a mock control and actin levels as controls is shown.

### Endogenous CYTIP becomes Ubiquitinated

**Figure 4:**
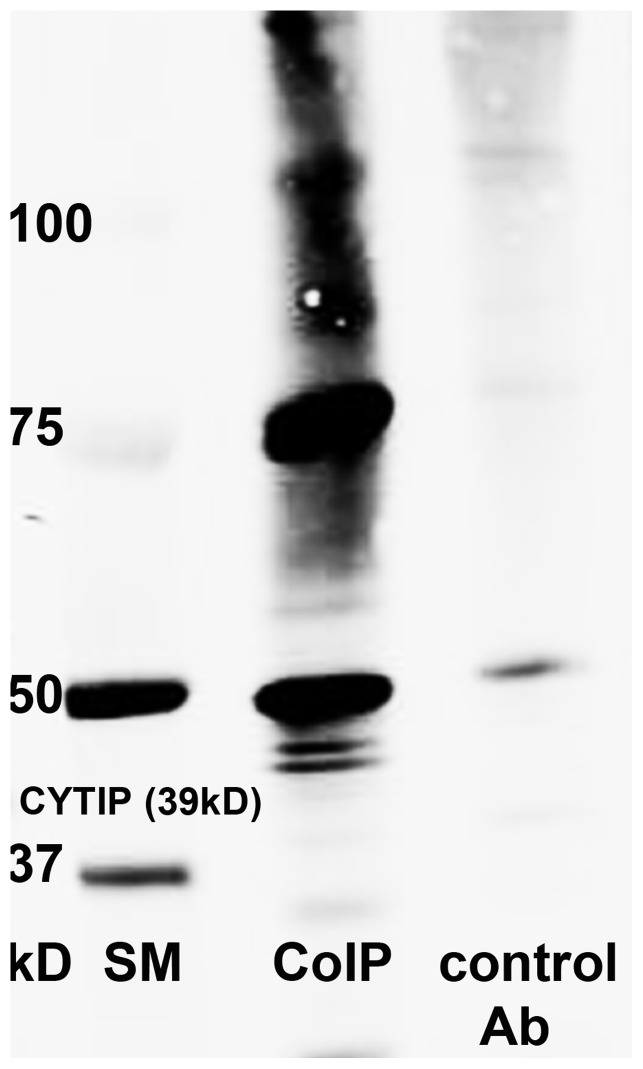
Co-immunoprecipitation of CYTIP with Ubiquitin. The immunocomplex obtained by incubating 2 mg of mature dendritic cells lysate with rat polyclonal anti CYTIP antibody (1A3) was analyzed by Western blot analyses with mouse anti ubiquitin and visualized with Alexa fluor 680 goat anti mouse antibody. Ubiquitination of the CYTIP precipitate is shown (Co-IP). As a control the precipitate obtained with rat isotype control is shown.

To gain further evidence for the hypothesis that CYTIP is taken to the proteasome for degradation by binding to SOCS-1, we investigated whether CYTIP is ubiquitinated. Ubiquitin is a ubiquitously expressed protein used to label proteins for degradation by the proteasome. We performed co-immunoprecipitation using 8 µg of a rat monoclonal anti CYTIP antibody or an isotype matched antibody as a control. In Western blot analysis with a mouse anti ubiquitin monoclonal antibody, ubiquitinated bands of approximately 50 kDa, 75 kDa, and two or three additional minor bands between 75 and 100 kDa were detected in the immunoprecipitate obtained with the anti CYTIP antibody but not with the isotype matched control antibody (Fig. 4). We assume that the bands consist of CYTIP with 2 (50 kDa), 4 (75 kDa) or more ubiquitin copies attached. This indicates that CYTIP is ubiquitinated in monocyte derived mature dendritic cells.

### Degradation of CYTIP is Inhibited by the Proteasome Inhibitor Bortezomib/Velcade®

**Figure 5:**
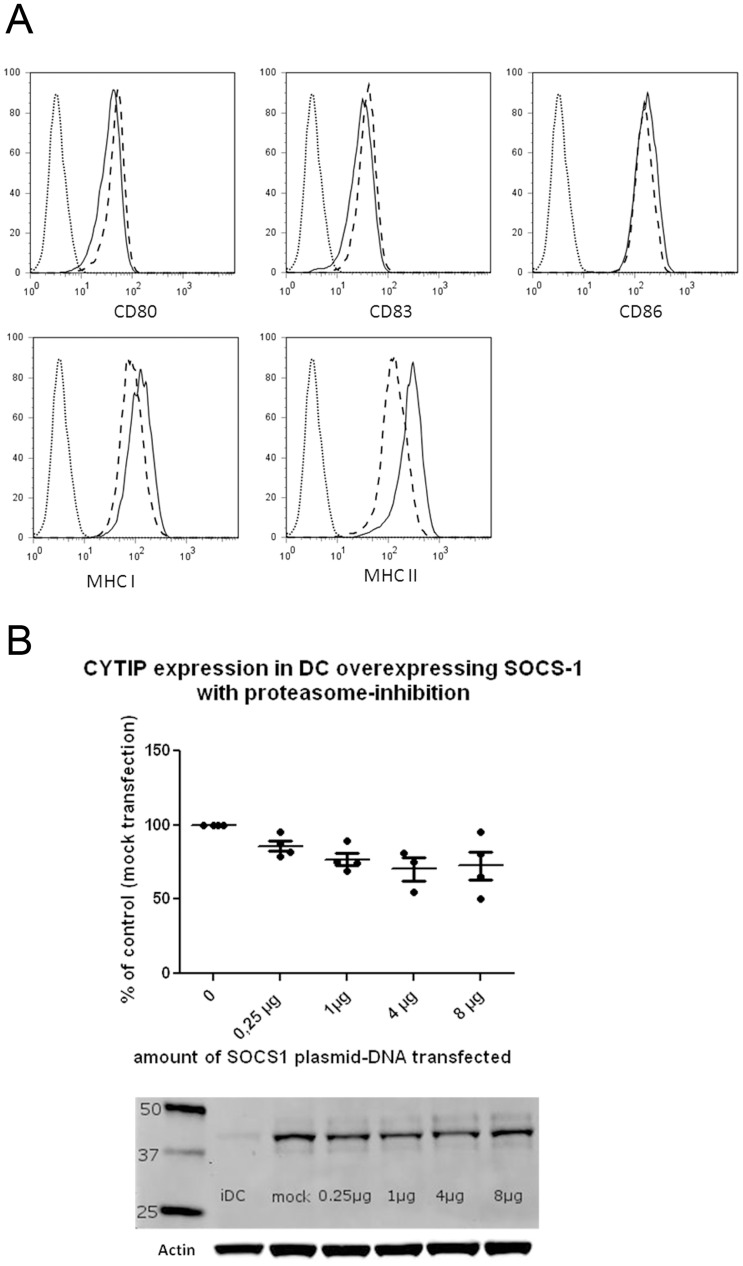
(A) FACS analysis of cell surface markers on untreated or 5 nM Bortezomib treated mature dendritic cells. Maturation marker expression (CD80, CD83 and CD86, MHC class I and MHC class II) on mature dendritic cells and mature dendritic cells treated with 5 nM Bortezomib were measured. Maturation markers show no difference when treated with 5 nM Bortezomib. Expression of MHC class I and MHC class II increase slightly in 5 nM Bortezomib treated dendritic cells. (B): Dendritic cells treated to inhibit the proteasome show stable CYTIP levels with SOCS-1 over expression. Mature dendritic cells treated with 5 nM Bortezomib and transfected with the indicated amounts of SOCS-1 encoding plasmid for 16 hours were harvested and protein lysates were prepared. 40 µg of lysates were applied to each lane and CYTIP levels (39 kDa) were detected by Western blot analysis. As a control, actin expression levels were determined. CYTIP expression levels correlated to actin expression in four independent experiments are shown. Dots represent CYTIP expression levels of the individual experiments in % of mock control. Lines are mean values. No significant decrease is obtained.

If SOCS-1 takes CYTIP to the proteasome for degradation treatment of monocyte dendritic cells with the proteasome inhibitor Bortezomib (Velcade®) should keep CYTIP levels constant when a SOCS-1 encoding plasmid is transfected in increasing concentrations. Treatment of dendritic cells with 7.5 nM Bortezomib has been shown to have an inhibitory effect on the maturation of dendritic cells while at 5 nM this effect was only seen as a tendency [16]. We therefore used 5 nM Bortezomib in our experiments to avoid inhibitory effects on dendritic cell maturation. Dendritic cells induced to mature and simultaneously treated with 5 nM Bortezomib indeed showed only marginal changes in the expression levels of the maturation markers CD80, CD83 and CD86. Expression of the MHC molecules (HLA-ABC and HLA-DR) was, however, increased, possibly because of an inhibition of their degradation by the proteasome (Fig. 5a). Dendritic cells were simultaneously matured and treated with 5 nM Bortezomib for 24 hours before transfection with the SOCS-1 encoding plasmid. CYTIP levels in Western blot analyses remained stable when dendritic cells were treated with Bortezomib (Fig. 5b). Statistical analysis using paired, two-tailed student’s t-test was performed on four independent experiments. CYTIP expression levels remained constant with the indicated amounts of SOCS-1 encoding plasmid used for transfection.

## Discussion

SOCS-1, the newly identified binding partner of CYTIP, belongs to a family of eight intracellular proteins which have various functions in different cell types. Several members of this family have been shown to regulate responses of immune cells to cytokines. Since SOCS proteins are also induced by cytokines they act in a classical negative feedback mechanism to inhibit cytokine signal transduction [17], [18]. The SOCS-1 molecule consists of 3 different domains, a kinase inhibitory region (KIR), a SH2 domain and a SOCS-box [17]. Through its KIR domain SOCS-1 can directly inhibit JAK tyrosine kinase activity. JAK tyrosine kinase is an important cytokine signaling molecule and the KIR domain of SOCS-1 is proposed to function as a pseudo substrate and is, thus, important for the suppression of cytokine signals. The SH2 domain binds to phosphorylated tyrosine in the JAK kinase activation loop and thus contributes to the inhibition of the JAK kinase activity of cytokine receptors. The SOCS-box domain of SOCS-1 is part of the E3 ubiquitin ligase complex [19]. SOCS-1 functions as an adapter between elongin BC complex, a component of the ubiquitin proteasome pathway, and SOCS-1 binding partners [18]. SOCS-1 deficient mice die early after birth because of severe systemic inflammation and aberrant activation of T cells, mainly as a result of uncontrolled cytokine signaling and production [20]. These multiple and important functions of SOCS-1 led us to further investigate the functional consequences of the interaction of CYTIP and SOCS-1. The expression pattern of CYTIP during the maturation of dendritic cells shows an increase up to 72 h and a decrease at later time points (Fig. 2). This is concordant with the possibility that SOCS-1 might take CYTIP to the proteasome for degradation. To evaluate this we followed the expression of CYTIP in dendritic cells with SOCS-1 over expression without (Fig. 3) and with blocked proteasome (Fig. 5). In consistency with the function of SOCS-1 to bind CYTIP for degradation we found decreased expression of CYTIP in dendritic cells over expressing SOCS-1 while the expression remains stable when in the same setting the proteasome is blocked. Thus, we provide evidence that SOCS-1 is involved in the degradation of CYTIP at later time points during maturation. The findings of Theodoridis et al [9] showing that after HSV infection of human monocyte derived dendritic cells CYTIP is quickly degraded, leading to impaired migration and T cell activation and of Shen et al. [21] showing a critical role for SOCS-1 in regulating the extent of antigen presentation by matured dendritic cells emphasize the importance of the newly identified interaction of these two proteins. Shen et. al. deduce from the data that SOCS-1 silenced dendritic cells might be capable of turning off regulatory T cells by enhancing dendritic cell maturation and the production of pro inflammatory cytokines and, thus, an important function of SOCS-1 in immune responses by down regulating signaling cascade molecules and cytokine pathways [21]. Combined with our finding that CYTIP is bound to SOCS-1 to get degraded by the proteasome these data provide a regulatory mechanism that allows dendritic cells to control the magnitude and duration of an immune response and emphasize the importance of both molecules in the control of immunity.
